# Enantioselective Drug Recognition by Drug Transporters

**DOI:** 10.3390/molecules23123062

**Published:** 2018-11-22

**Authors:** Yuichi Uwai

**Affiliations:** School of Pharmacy, Aichi Gakuin University, Nagoya 464-8650, Japan; yuuwai@dpc.agu.ac.jp; Tel.: +81-52-757-6785

**Keywords:** drug transporter, enantioselectivity, transport, inhibition, pharmacokinetics

## Abstract

Drug transporters mediate the absorption, tissue distribution, and excretion of drugs. The cDNAs of P-glycoprotein, multidrug resistance proteins (MRPs/ABCC), breast cancer resistance protein (BCRP/ABCG2), peptide transporters (PEPTs/SLC15), proton-coupled folate transporters (PCFT/SLC46A1), organic anion transporting polypeptides (OATPs/SLCO), organic anion transporters (OATs/SLC22), organic cation transporters (OCTs/SLC22), and multidrug and toxin extrusions (MATEs/SLC47) have been isolated, and their functions have been elucidated. Enantioselectivity has been demonstrated in the pharmacokinetics and efficacy of drugs, and is important for elucidating the relationship with recognition of drugs by drug transporters from a chiral aspect. Enantioselectivity in the transport of drugs by drug transporters and the inhibitory effects of drugs on drug transporters has been summarized in this review.

## 1. Introduction

Many drugs are chiral, and each enantiomer may exhibit specific therapeutic efficacy. For example, several nonsteroidal antiinflammatory drugs (NSAIDs) have an asymmetric carbon in their chemical structures, and the (*S*)-enantiomers exhibit stronger inhibitory potencies against cyclooxygenases [1]. The (*S*)-enantiomer of naproxen is commercially available. We examined the enantioselective effects of flurbiprofen on the disposition of lithium in rats, and demonstrated that the (*S*)-enantiomer, but not the (*R*)-enantiomer, decreased the renal clearance of lithium with the impairment of renal function [2]. The anticoagulant drug warfarin is also chiral. Warfarin is orally administered as the racemate and exhibits enantioselectivity not only in its pharmacological effects, but also in its pharmacokinetics. (*S*)-Warfarin has greater anticoagulant potency than the (*R*)-enantiomer [3]. (*S*)-Warfarin is metabolized by cytochrome P450 (CYP) 2C9, and the metabolism of the (*R*)-enantiomer is mediated by CYP1A2 and CYP3A4 [4]. The development of drugs with enantioselective pharmacokinetic properties, such as esomeprazole, has recently been accomplished. This proton pump inhibitor is the (*S*)-enantiomer of omeprazole, which is the racemate. Both enantiomers exert identical effects on H^+^, K^+^-ATPase [5], but exhibit different pharmacokinetic characteristics. The clearance of esomeprazole is lower than that of the (*R*)-enantiomer [6]. Furthermore, the metabolism of the (*R*)-enantiomer is mainly mediated by CYP2C19, which exhibits a genetic polymorphism, and the contribution of CYP2C19 to the metabolism of esomeprazole is negligible [6].

In 1988, CYP2D6 was the first cytochrome P450 enzyme whose cDNA was identified [7]. The cDNAs of organic ion transporters have been identified since the 1990s. These findings promoted research on the function and expression of drug transporters. Drug transporters are expressed in the plasma membranes of a number of cells including enterocytes, hepatocytes, brain microvascular endothelial cells, and renal epithelial cells, and facilitate the transport of drugs across the plasma membrane. Drug transporters are now known to play important roles in the absorption, tissue distribution, and excretion of drugs. Previous studies reported enantioselective drug transport by drug transporters. Furthermore, the enantioselective inhibitory effects of drugs on drug transporters has been demonstrated. 

**Figure 1:**
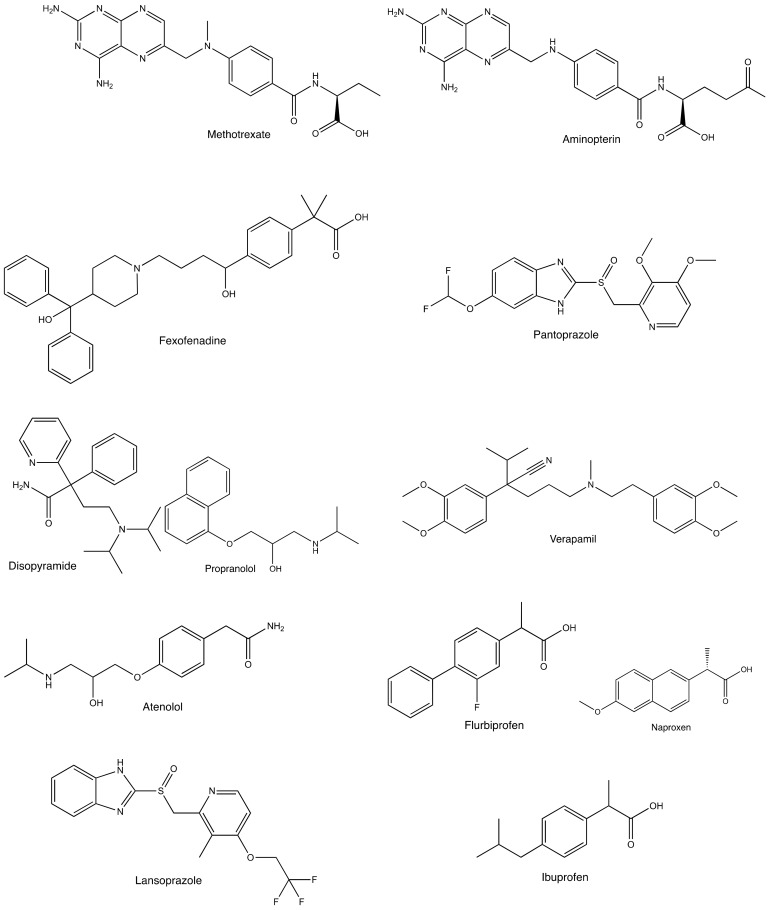
Chemical structures of chiral drugs described in this review.

Representative drug transporters functioning in the intestinal absorption, biliary excretion, and renal tubular secretion of drugs have been summarized in this review. The interactions between chiral drugs and drug transporters have also been discussed. Figure 1 shows the chemical structures of the chiral drugs described herein. 

## 2. Role of Drug Transporters in the Intestinal Absorption, Biliary Excretion, and Renal Tubular Secretion of Drugs

### 2.1. Intestinal Drug Absorption by Drug Transporters

**Figure 2:**
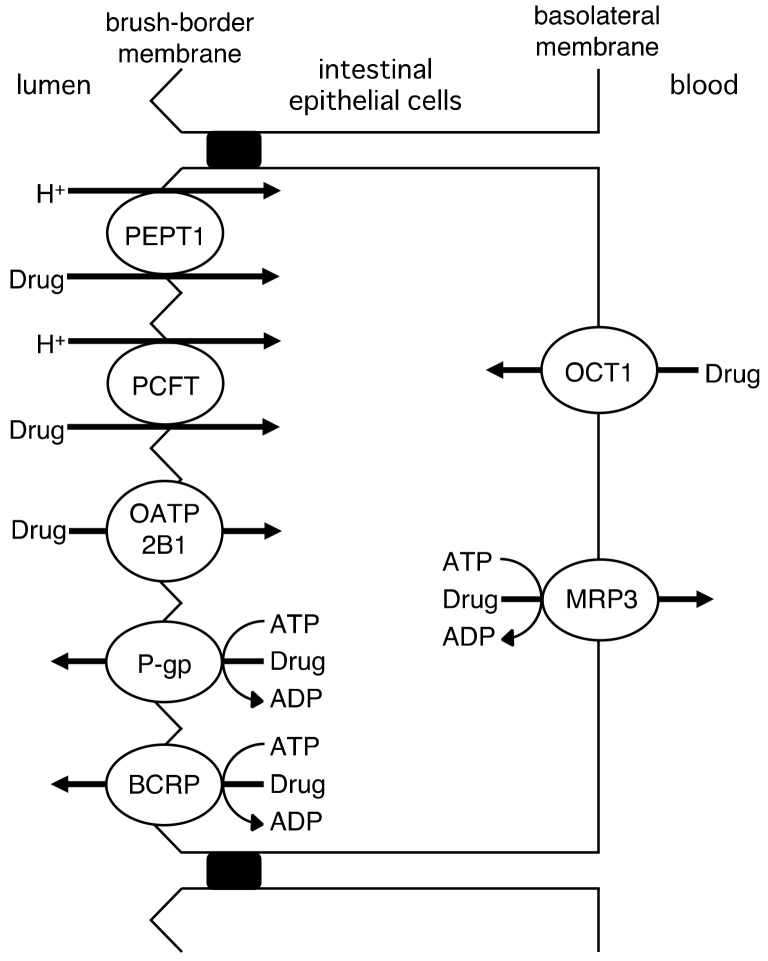
Drug transporters in the small intestine.

Drugs are absorbed by the small intestine after their oral administration. Figure 2 shows drug transporters that are involved in the intestinal absorption of drugs. Peptide transporter PEPT1 (SLC15A1) is one of the most extensively studied drug transporters in the small intestine. It is expressed in the brush-border membrane of intestinal epithelial cells and is responsible for the uptake of dipeptides and tripeptides from the lumen using an inward H^+^-electrochemical gradient [8]. PEPT1 recognizes peptide-like β-lactam antibiotics that are orally administered [8]. PEPT1 is used as a target molecule for improving the absorption of poorly absorbed drugs. Valganciclovir, the valine ester prodrug of ganciclovir, was developed to enhance the low bioavailability of ganciclovir, and the mechanism responsible for improved absorption was identified as drug recognition by PEPT1 [9]. 

The proton-coupled folate transporter PCFT (SLC46A1) is localized to the brush-border membrane of enterocytes, and mediates the absorption of folate [10]. PCFT recognizes the antifolates, methotrexate and aminopterin, as its substrates [11,12]. Aminopterin has not yet been approved as a medicine, and methotrexate is used in the treatment of neoplasia and rheumatoid arthritis. PCFT is considered to play a role in the intestinal absorption of orally administered methotrexate [13]. 

The organic anion transporting polypeptide OATP2B1 (SLCO2B1), which is expressed in the brush-border membrane of intestinal epithelial cells, is crucially involved in the uptake of fexofenadine, celiprolol, and montelukast from the lumen [14,15]. 

P-glycoprotein (P-gp; ABCB1) and the breast cancer resistance protein BCRP (ABCG2) are ATP-binding cassette (ABC) transporter proteins that are expressed in the brush-border membrane of enterocytes. These transporters are efflux pumps. P-gp transports various types of drugs, including anticancer agents, antihypertensive agents, antiarrhythmics, antidepressants, antimicrobial agents, anti-human immunodeficiency virus agents, anticonvulsants, antiemetics, immunosuppressants, neuroleptics, and opioids, and there is a broad range of overlapping substrate specificities for CYP3A4 and P-gp [16]. BCRP has also been shown to transport anticancer agents [17]. The tyrosine kinase inhibitors, imatinib, gefitinib, and nilotinib, have also been identified as substrates of BCRP [18,19,20]. P-gp and BCRP prevent drug absorption in the intestine.

In the basolateral membrane, the organic cation transporter OCT1 (SLC22A1) and the multidrug resistance protein MRP3 (ABCC3) mediate drug transport [21]. In OCT1 knockout mice, the intestinal excretion of the typical substrate, tetraethylammonium, was reduced [22]. The serosal efflux of the glucuronide conjugates of 7-ethyl-10-hydroxycamptothecin (SN-38: the active metabolite of irinotecan) and acetaminophen in the jejunal everted sacs was decreased in Mrp3 knockout mice [23].

### 2.2. Hepatic Transport of Drugs by Drug Transporters

**Figure 3:**
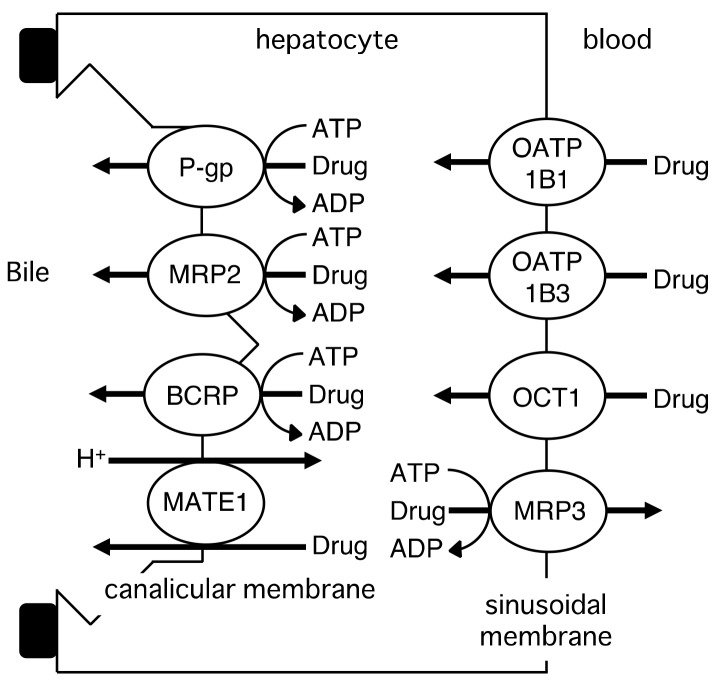
Drug transporters in the liver.

Drug transporters in the plasma membrane of hepatocytes are shown in Figure 3. The OATP family members OATP1B1 (SLCO1B1) and OATP1B3 (SLCO1B3) mediate the hepatic uptake of various drugs, such as HMG-CoA reductase inhibitors, angiotensin II receptor antagonists, nateglinide, asunaprevir, pemafibrate, and bosentan, from the circulation [14,24]. OCT1 functions in the sinusoidal uptake of organic cations, including metformin, tropisetron, sumatriptan and fenoterol, from the circulation [25,26,27,28]. P-gp, MRP2 (ABCC2), BCRP, and multidrug and toxin extrusion MATE1 (SLC47A1), which are expressed in the canalicular membrane, mediate the efflux of drugs into bile [24]. MRP2, a member of the ABC family of transporters, excretes monoglucuronosyl bilirubin and monoglucuronosyl bilirubin into bile, and genetic mutations in MRP2 cause Dubin-Johnson syndrome, an autosomal recessive disease characterized by conjugated hyperbilirubinemia [29]. MRP3 is localized to the sinusoidal membrane of hepatocytes [30]. Kitamura et al. reported decreased plasma levels and increased clearance for the biliary excretion of methotrexate in MRP3 knockout mice, suggesting that MRP3 transports methotrexate from hepatocytes into plasma [31]. 

### 2.3. Renal Tubular Secretion of Drugs

**Figure 4:**
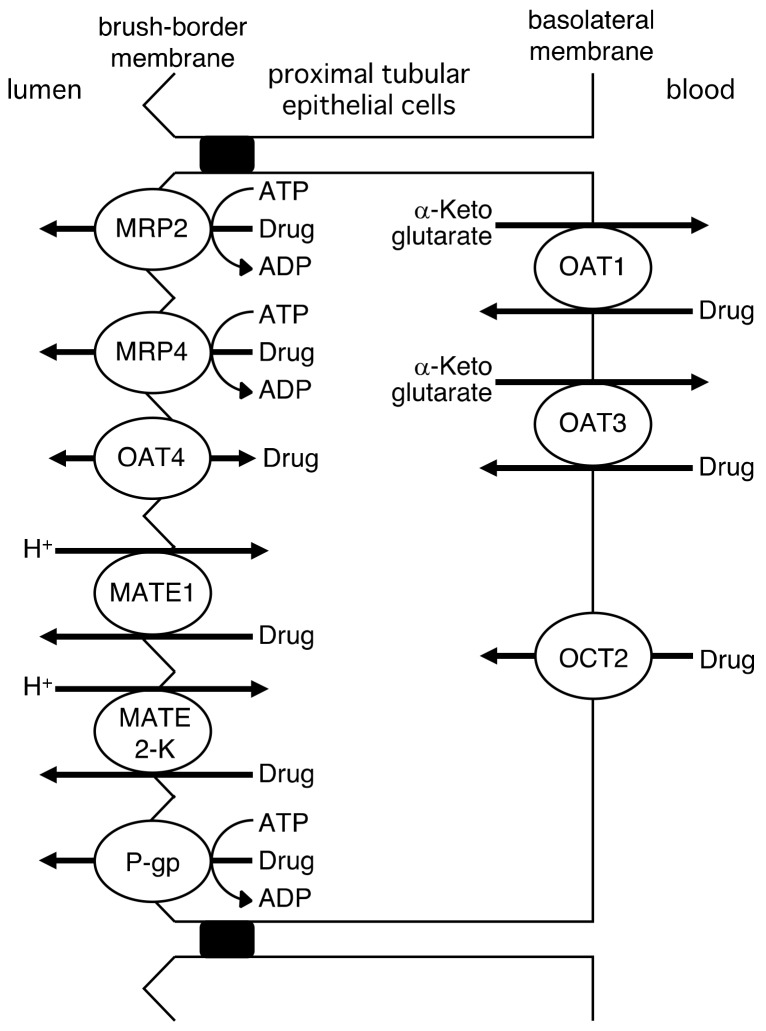
Drug transporters mediating renal tubular secretion drugs.

The renal tubular secretion of drugs is mediated by drug transporters, as shown in Figure 4. Organic anion and cation transport systems are present in renal proximal epithelial cells. The organic anion transporter system is involved in the tubular secretion of anionic drugs, including anticancer agents, β-lactam antibiotics, antivirals, and diuretics [24,32]. The organic anion transporters OAT1 (SLC22A6) and OAT3 (SLC22A8) are responsible for the basolateral uptake of anionic drugs from the circulation via an exchange with intracellular α-ketoglutarate, an intermediate in the Krebs cycle. Information on the drug transporters responsible for mediating the efflux of drugs via the brush-border membrane of proximal epithelial cells is more limited than that on basolateral transporters. Studies using rat renal brush-border membrane vesicles indicated that a potential-sensitive organic anion transporter and an anion/organic anion exchange transporter function [33,34,35]. However, their genes have not yet been identified. Previous studies reported reductions in the urinary excretion of adefovir, tenofovir, hydrochlorothiazide, furosemide, ceftizoxime, and cefazolin in MRP4 knockout mice [36,37,38]. Based on these findings, MRP4 has been suggested to play an important role in drug transport in the brush-border membrane. MRP2 and OAT4 are known to be expressed in the brush-border membrane of proximal epithelial cells [39,40]. Although their interactions with drugs have been investigated in in vitro experiments, the roles of MRP2 and OAT4 in the tubular transport of drugs currently remain unclear. 

The organic cation transport system consists of the uptake type of OCT2 (SLC22A2) and the efflux modes of MATE1 and MATE2-K (SLC47A2) [24,32,41]. Drug transport by OCT2 is electrogenic, and is driven by an internal negative membrane potential. The efflux of drugs by MATE1 and MATE2-K is dominated by a H^+^/organic cation antiport, which involves electroneutral transport [41]. Cationic drugs, including cimetidine, metformin, procainamide, memantine, and amantadine, are secreted into urine by the organic cation transport system [32].

P-gp functions in the renal tubular secretion of the cardiac glycoside, digoxin [42]. Although a wide variety of drugs are transported by P-gp [16], most are not recovered into urine. The substrate drugs of P-gp may be reabsorbed by the distal tubules via simple diffusion after tubular secretion.

## 3. Enantioselective Drug Transport by Drug Transporters

### 3.1. Enantioselective Transport of Antifolates by PCFT

Previous studies reported enantioselectivity in drug transport by drug transporters, with PCFT as a representative. The antifolates methotrexate and aminopterin have an asymmetric carbon in their structures (Figure 1), and their enantioselective transport was examined. Narawa et al. constructed stably transfected human embryonic kidney cells 293 (HEK293 cells) expressing PCFT, and conducted a methotrexate uptake experiment using these cells [43]. Menter et al. examined aminopterin transport using Chinese hamster ovary (CHO) cells expressing PCFT [12]. The transport of both antifolates showed enantioselectivity, and kinetic parameters were summarized in Table 1. The (*S*)-enantiomers were found to have greater affinity to PCFT than each (*R*)-enantiomer. Menter et al. performed a pharmacokinetic study on aminopterin in dogs and patients with psoriasis, and the findings obtained revealed a correlation with enantioselective absorption and in vitro findings [12].

### 3.2. Enantioselectivity in the Pharmacokinetics of Fexofenadine and Its Transport by OATP2B1

Fexofenadine, a histamine H_1_-receptor antagonist, has non-sedative properties that have been attributed to the restriction of its brain penetration by P-gp [44]. Enantioselectivity has been reported in its pharmacokinetics. Miura et al. demonstrated that the maximum plasma concentration and area under the plasma concentration-time curve of (*R*)-fexofenadine were higher than those of the (*S*)-enantiomer after the single oral administration of racemic fexofenadine to healthy volunteers [45]. Sakugawa et al. examined effect of verapamil, an inhibitor of P-gp, on the disposition of each enantiomer of fexofenadine in healthy volunteers, and suggested that the other mechanisms in addition to P-gp contribute to the stereoselective pharmacokinetics of fexofenadine [46]. To my knowledge, there is no reports representative of enantioselective transport of fexofenadine by P-gp from in vitro experiments. Most of the dosage of fexofenadine administered is excreted into urine in its unmetabolized form, and various drug transporters have been shown to contribute to its pharmacokinetics. The OATP family members, OATP1A2, OATP1B1, OATP1B3, and OATP2B1, were proposed to be responsible for the intestinal uptake of fexofenadine or its distribution into the liver [47,48,49,50,51,52]. MRP2 and the bile salt export pump BSEP (ABCB11) mediate the efflux of fexofenadine from hepatocytes into bile, while MRP3 transports it into plasma [52,53]. OAT3 plays a role in the renal tubular uptake of fexofenadine, and MATE1 contributes to its efflux into urine [54,55]. At least one of the drug transporters described above may be responsible for the enantioselective pharmacokinetics of fexofenadine. The enantioselective transport of fexofenadine has only been reported to occur by OATP2B1. Akamine et al. found greater uptake amounts of (*R*)-fexofenadine in *Xenopus* oocytes injected with OATP2B1 cRNA than that of the (*S*)-enantiomer [56]. They also demonstrated that apple juice decreased the absorption of fexofenadine orally administered to healthy volunteers, and that the juice inhibited its transport by OATP2B1 [56]. Accordingly, OATP2B1 appears to mediate the enantioselective absorption of fexofenadine by the small intestine. Akamine et al. did not describe the kinetic parameters of the transport of enantiomers by OATP2B1. The renal clearance of (*S*)-fexofenadine was higher than that of the (*R*)-enantiomer [45,56], whereas Kusuhara et al. reported no enantioselective transport of fexofenadine by OAT3 and MATE1 [57]. In addition, they showed the similar transport of both enantiomers by OATP1B3 [57]. Unidentified drug transporter(s) may be responsible for the enantioselective disposition of fexofenadine. 

### 3.3. Enantioselective Secretion of Pantoprazole into Milk by BCRP

Enantioselective drug transport was demonstrated with the combination of pantoprazole and BCRP. BCRP affects the absorption, distribution, and excretion of drugs, and BCRP actively secretes xenobiotics, including drugs and carcinogens, and riboflavin into milk [58,59,60]. Wang et al. showed the greater accumulation of (−)-pantoprazole in the milk of lactating rats infused with racemic pantoprazole than that of (+)-pantoprazole [61], and the higher affinity of the (−)-enantiomer with BCRP [62]. 

## 4. Enantioselective Inhibitory Effects of Drugs on Drug Transporters

### 4.1. Enantioselectivity in Inhibitory Effects of Drugs on OCT1, and Binding Affinities

Enantioselectivity was shown in the inhibitory effect of disopyramide and propranolol on OCT1. The (*R*)-disopyramide inhibited the uptake of tetraethylammonium by HeLa cells expressing OCT1 more strongly than the (*S*)-enantiomer [63], and also for propanol the inhibitory effect of (*S*)-enantiomer is stronger than for the (*R*)-enantiomer [64]. The IC_50_ values of each enantiomer were described in Table 2. 

Moaddel et al. studied the binding of drugs to OCT1 with a liquid chromatography stationary phase containing immobilized membranes obtained from a cell line that expresses OCT1, and estimated their binding affinities using frontal displacement chromatography with [^3^H]1-methyl 4-phenyl pyridinium as the marker ligand. The significant enantioselectivity on the binding to OCT1 was recognized in verapamil, atenolol, and propranolol [64,65]. (*R*)-Verapamil, (*S*)-atenolol, and (*S*)-propranolol showed the higher affinities than each enantiomer. In Table 3, their K_d_ values are summarized.

### 4.2. Enantioselective Inhibitory Effects of NSAIDs and Lansoprazole on OAT1 and OAT3

The inhibition of renal organic anion transporters leads to the delayed elimination of their substrates from the circulation. NSAIDs interfere with the renal excretion of methotrexate, and this combination is a representative among drug interactions via OAT1 and OAT3. The interaction is fatal when high-dose methotrexate therapy is given to a patient [66,67]. Previous studies demonstrated the inhibitory effects of NSAIDs, including cyclooxygenase-2 inhibitors, on methotrexate uptake by OAT1 and OAT3 [68,69,70,71,72]. Some NSAIDs are chiral (Figure 1). We conducted a drug transport experiment using the *Xenopus* oocyte expression system in order to examine enantioselectivity in the inhibition of OAT1 and OAT3 by NSAIDs [73]. The findings obtained showed the stronger inhibitory effects of the (*S*)-enantiomers of flurbiprofen, ibuprofen, and naproxen on the transport of *p*-aminohippurate and methotrexate by OAT1 than that of each (*R*)-enantiomer. The inhibitory mechanisms of flurbiprofen were investigated, and both enantiomers were found to competitively inhibit OAT1. Enantioselective differences were not observed in the inhibition of OAT3. 

Proton pump inhibitors also interact with methotrexate [74,75]; OAT1 and OAT3 were found to be inhibited by omeprazole, lansoprazole, pantoprazole, rabeprazole, and esomeprazole [76,77]. Chirality exists in the structures of proton pump inhibitors (Figure 1), and enantioselectivity was examined in the inhibition of OAT1 and OAT3 by lansoprazole [78]. The inhibitory effects of (*S*)-lansoprazole on the transport of estrone sulfate, methotrexate, and pemetrexed by OAT3 were stronger than those of the (*R*)-enantiomer. Furthermore, enantioselectivity was not recognized against OAT1. Enantioselectivity has been demonstrated in the pharmacokinetics of lansoprazole. The slower elimination of the (*R*)-enantiomer from plasma has been reported in healthy subjects [79]. The faster metabolism of (*S*)-lansoprazole by human liver microsomes was also shown [80]. This information is useful when considering drug interactions between lansoprazole and the substrate drugs of OAT3.

### 4.3. Enantioselective Inhibitory Effects of NSAIDs on MRP2 and MRP4

The enantioselective inhibitory effects of NSAIDs on MRP2 and MRP4 have been examined. Kawase et al. performed uptake experiments on methotrexate using MRP2- and MRP4-expressing inside-out vesicles, and showed the stronger inhibition of MRP2 by the (*S*)-enantiomers of flurbiprofen, ibuprofen, and naproxen than by each (*R*)-enantiomer [81]. In the case of MRP4, the (*R*)-enantiomers exerted strong inhibitory effects. 

Table 4 summarizes the IC_50_ values of each enantiomer of flurbiprofen, ibuprofen, naproxen, and lansoprazole described above. The most prominent enantioselective difference was noted in the inhibition of MRP2 by naproxen. 

## 5. Conclusions

Enantioselectivity has been demonstrated in the transport of methotrexate and aminopterin by PCFT, pantoprazole by BCRP, and fexofenadine by OATP2B1. Enantioselective differences were reported in the inhibitory effects of flurbiprofen, ibuprofen, and naproxen on OAT1, MRP2, and MRP4, and of lansoprazole on OAT3. The number of studies on enantioselective drug recognition by drug transporters is markedly smaller than those on drug metabolism enzymes. In research on enantioselectivity, data on drug metabolism accumulate, and Niwa et al. performed meta-analysis based on the reported values regarding the Michaelis-Menten constant, maximal velocity, intrinsic clearance, and inhibition constants [82]. They considered that there is a limited number of reports regarding stereoselective inhibition and induction in vitro [82]. Because drug transporters are also involved in drug interactions, it is desired to pay attention to in enantioselectivity in inhibition as well as in substrate recognition of drug transporters. 

Computational methods such as quantitative structure-activity relationship (QSAR) and pharmacophore approaches have become more widely applied to assess interactions between drugs and drug transporters and predictions for substrates and inhibitors for several transporters were described [83]. Because ignoring stereoselectivity reduces the accuracy of the QSAR and modelling analysis, stereoselectivity should become a key aspect of the modelling of interactions between drugs and drug transporters [84].

In the future, research on drug transporters from the chiral aspect of drugs will provide important insights into pharmacokinetics, pharmacodynamics, and drug toxicity.

## Figures and Tables

**Table 1:** Kinetic parameters of the PCFT-mediated transport of methotrexate and aminopterin.

Drug	K_m_ or K_t_ (*R*)-enantiomer	K_m_ or K_t_ (*S*)-enantiomer	K_m_ or K_t_ (*R*)/(*S*)	V_max_ (*R*)-enantiomer	V_max_ (*S*)-enantiomer	V_max_ (*R*)/(*S*)	Ref.
Methotrexate	211 µM	4.98 µM	42.4	909 pmol/mg/min	891 pmol/mg/min	1.02	[43]
Aminopterin	15.0 µM	0.69 µM	21.7	42.9 pmol/mg/2 min	68.8 pmol/mg/2 min	0.624	[12]

**Table 2:** IC_50_ values of each enantiomer of disopyramide and propranolol for OCT1.

Drug	IC_50_ Value (µM) (*R*)-enantiomer	IC_50_ Value (µM) (*S*)-enantiomer	(*R*)/(*S*)	Ref.
Disopyramide	15.4	29.9	0.515	[63]
Propranolol	41.7	15.1	2.76	[64]

**Table 3:** K_d_ values of each enantiomer of verapamil, atenolol, and propranolol for OCT1.

Drug	K_d_ Value (µM) (*R*)-enantiomer	K_d_ Value (µM) (*S*)-enantiomer	(*R*)/(*S*)	Ref.
Verapamil	0.05	3.46	0.0145	[65]
Atenolol	0.98	0.46	2.13	[64]
Propranolol	2.85	0.95	3.00	[64]

**Table 4:** IC_50_ values of each enantiomer of NSAIDs and lansoprazole for OAT1, OAT3, MRP2, and MRP4.

Drug	Transporter	Substrate	IC_50_ Value (µM) (*R*)-Enantiomer	IC_50_ Value (µM) (*S*)-Enantiomer	(*R*)/(*S*)	Ref.
Flurbiprofen	OAT1	*p*-aminohippurate	2.35	0.615	3.82	[73]
	OAT3	estrone sulfate	2.13	1.80	1.18	[73]
	MRP2	methotrexate	133	58.4	2.28	[81]
	MRP4	methotrexate	10.6	37.2	0.285	[81]
Ibuprofen	OAT1	*p*-aminohippurate	6.14	2.84	2.16	[73]
	OAT3	estrone sulfate	2.04	1.20	1.70	[73]
	MRP2	methotrexate	303	139	2.18	[81]
	MRP4	methotrexate	129	267	0.483	[81]
Naproxen	OAT1	*p*-aminohippurate	5.26	1.93	2.73	[73]
	OAT3	estrone sulfate	8.09	6.79	1.19	[73]
	MRP2	methotrexate	510	7.11	71.7	[81]
	MRP4	methotrexate	8.06	49.8	0.162	[81]
Lansoprazole	OAT1	*p*-aminohippurate	43.8	33.6	1.30	[78]
	OAT3	estrone sulfate	1.75	0.61	2.87	[78]

## References

[1] Carabaza A., Cabré F., Rotllan E., Gómez M., Gutiérrez M., García M.L., Mauleón D. (1996). Stereoselective inhibition of inducible cyclooxygenase by chiral nonsteroidal antiinflammatory drugs. J. Clin. Pharmacol..

[2] Uwai Y., Matsumoto M., Kawasaki T., Nabekura T. (2017). Enantioselective effect of flurbiprofen on lithium disposition in rats. Pharmacology.

[3] Park B.K. (1988). Warfarin: Metabolism and mode of action. Biochem. Pharmacol..

[4] Kaminsky L.S., Zhang Z.Y. (1997). Human P450 metabolism of warfarin. Pharmacol. Ther..

[5] Kendall M.J. (2003). Review article: Esomeprazole—The first proton pump inhibitor to be developed as an isomer. Aliment. Pharmacol. Ther..

[6] Andersson T., Hassan-Alin M., Hasselgren G., Röhss K., Weidolf L. (2001). Pharmacokinetic studies with esomeprazole, the (*S*)-isomer of omeprazole. Clin. Pharmacokinet..

[7] Gonzalez F.J., Vilbois F., Hardwick J.P., McBride O.W., Nebert D.W., Gelboin H.V., Meyer U.A. (1988). Human debrisoquine 4-hydroxylase (P450IID1): cDNA and deduced amino acid sequence and assignment of the CYP2D locus to chromosome 22. Genomics.

[8] Terada T., Inui K. (2004). Peptide transporters: Structure, function, regulation and application for drug delivery. Curr. Drug Metab..

[9] Sugawara M., Huang W., Fei Y.J., Leibach F.H., Ganapathy V., Ganapathy M.E. (2000). Transport of valganciclovir, a ganciclovir prodrug, via peptide transporters PEPT1 and PEPT2. J. Pharm. Sci..

[10] Qiu A., Jansen M., Sakaris A., Min S.H., Chattopadhyay S., Tsai E., Sandoval C., Zhao R., Akabas M.H., Goldman I.D. (2006). Identification of an intestinal folate transporter and the molecular basis for hereditary folate malabsorption. Cell.

[11] Inoue K., Nakai Y., Ueda S., Kamigaso S., Ohta K.Y., Hatakeyama M., Hayashi Y., Otagiri M., Yuasa H. (2008). Functional characterization of PCFT/HCP1 as the molecular entity of the carrier-mediated intestinal folate transport system in the rat model. Am. J. Physiol. Gastrointest. Liver Physiol..

[12] Menter A., Thrash B., Cherian C., Matherly L.H., Wang L., Gangjee A., Morgan J.R., Maeda D.Y., Schuler A.D., Kahn S.J. (2012). Intestinal transport of aminopterin enantiomers in dogs and humans with psoriasis is stereoselective: Evidence for a mechanism involving the proton-coupled folate transporter. J. Pharmacol. Exp. Ther..

[13] Inoue K., Yuasa H. (2014). Molecular basis for pharmacokinetics and pharmacodynamics of methotrexate in rheumatoid arthritis therapy. Drug Metab. Pharmacokinet..

[14] Shitara Y., Maeda K., Ikejiri K., Yoshida K., Horie T., Sugiyama Y. (2013). Clinical significance of organic anion transporting polypeptides (OATPs) in drug disposition: Their roles in hepatic clearance and intestinal absorption. Biopharm. Drug Dispos..

[15] Tamai I., Nakanishi T. (2013). OATP transporter-mediated drug absorption and interaction. Curr. Opin. Pharmacol..

[16] Zhou S.F. (2008). Structure, function and regulation of P-glycoprotein and its clinical relevance in drug disposition. Xenobiotica.

[17] Mao Q., Unadkat J.D. (2015). Role of the breast cancer resistance protein (BCRP/ABCG2) in drug transport—An update. AAPS J..

[18] Burger H., van Tol H., Boersma A.W., Brok M., Wiemer E.A., Stoter G., Nooter K. (2004). Imatinib mesylate and nilotinib (AMN107) exhibit high-affinity interaction with ABCG2 on primitive hematopoietic stem cells. Blood.

[19] Elkind N.B., Szentpétery Z., Apáti A., Ozvegy-Laczka C., Várady G., Ujhelly O., Szabó K., Homolya L., Váradi A., Buday L. (2005). Multidrug transporter ABCG2 prevents tumor cell death induced by the epidermal growth factor receptor inhibitor Iressa (ZD1839, Gefitinib). Cancer Res..

[20] Brendel C., Scharenberg C., Dohse M., Robey R.W., Bates S.E., Shukla S., Ambudkar S.V., Wang Y., Wennemuth G., Burchert A. (2007). Imatinib mesylate and nilotinib (AMN107) exhibit high-affinity interaction with ABCG2 on primitive hematopoietic stem cells. Leukemia.

[21] Giacomini K.M., Huang S.M., Tweedie D.J., Benet L.Z., Brouwer K.L., Chu X., Dahlin A., Evers R., Fischer V., Hillgren K.M. (2010). Membrane transporters in drug development. Nat. Rev. Drug Discov..

[22] Jonker J.W., Wagenaar E., Mol C.A., Buitelaar M., Koepsell H., Smit J.W., Schinkel A.H. (2001). Reduced hepatic uptake and intestinal excretion of organic cations in mice with a targeted disruption of the organic cation transporter 1 (*Oct1* [*Slc22a1*]) gene. Mol. Cell. Biol..

[23] Kitamura Y., Kusuhara H., Sugiyama Y. (2010). Functional characterization of multidrug resistance-associated protein 3 (Mrp3/*Abcc3*) in the basolateral efflux of glucuronide conjugates in the mouse small intestine. J. Pharmacol. Exp. Ther..

[24] Kusuhara H., Sugiyama Y. (2009). In vitro-in vivo extrapolation of transporter-mediated clearance in the liver and kidney. Drug Metab. Pharmacokinet..

[25] Wang D.S., Jonker J.W., Kato Y., Kusuhara H., Schinkel A.H., Sugiyama Y. (2002). Involvement of organic cation transporter 1 in hepatic and intestinal distribution of metformin. J. Pharmacol. Exp. Ther..

[26] Tzvetkov M.V., Saadatmand A.R., Bokelmann K., Meineke I., Kaiser R., Brockmöller J. (2012). Effects of OCT1 polymorphisms on the cellular uptake, plasma concentrations and efficacy of the 5-HT_3_ antagonists tropisetron and ondansetron. Pharmacogenomics J..

[27] Matthaei J., Kuron D., Faltraco F., Knoch T., Dos Santos Pereira J.N., Abu Abed M., Prukop T., Brockmöller J., Tzvetkov M.V. (2016). OCT1 mediates hepatic uptake of sumatriptan and loss-of-function OCT1 polymorphisms affect sumatriptan pharmacokinetics. Clin. Pharmacol. Ther..

[28] Tzvetkov M.V., Matthaei J., Pojar S., Faltraco F., Vogler S., Prukop T., Seitz T., Brockmöller J. (2018). Increased systemic exposure and stronger cardiovascular and metabolic adverse reactions to fenoterol in individuals with heritable OCT1 deficiency. Clin. Pharmacol. Ther..

[29] Toh S., Wada M., Uchiumi T., Inokuchi A., Makino Y., Horie Y., Adachi Y., Sakisaka S., Kuwano M. (1999). Genomic structure of the canalicular multispecific organic anion-transporter gene (MRP2/cMOAT) and mutations in the ATP-binding-cassette region in Dubin-Johnson syndrome. Am. J. Hum. Genet..

[30] König J., Rost D., Cui Y., Keppler D. (1999). Characterization of the human multidrug resistance protein isoform MRP3 localized to the basolateral hepatocyte membrane. Hepatology.

[31] Kitamura Y., Hirouchi M., Kusuhara H., Schuetz J.D., Sugiyama Y. (2008). Increasing systemic exposure of methotrexate by active efflux mediated by multidrug resistance-associated protein 3 (mrp3/abcc3). J. Pharmacol. Exp. Ther..

[32] Ivanyuk A., Livio F., Biollaz J., Buclin T. (2017). Renal drug transporters and drug interactions. Clin. Pharmacokinet..

[33] Martinez F., Manganel M., Montrose-Rafizadeh C., Werner D., Roch-Ramel F. (1990). Transport of urate and *p*-aminohippurate in rabbit renal brush-border membranes. Am. J. Physiol..

[34] Ohoka K., Takano M., Okano T., Maeda S., Inui K., Hori R. (1993). *p*-Aminohippurate transport in rat renal brush-border membranes: A potential-sensitive transport system and an anion exchanger. Biol. Pharm. Bull..

[35] Inui K.I., Masuda S., Saito H. (2000). Cellular and molecular aspects of drug transport in the kidney. Kidney Int..

[36] Imaoka T., Kusuhara H., Adachi M., Schuetz J.D., Takeuchi K., Sugiyama Y. (2007). Functional involvement of multidrug resistance-associated protein 4 (MRP4/ABCC4) in the renal elimination of the antiviral drugs adefovir and tenofovir. Mol. Pharmacol..

[37] Hasegawa M., Kusuhara H., Adachi M., Schuetz J.D., Takeuchi K., Sugiyama Y. (2007). Multidrug resistance-associated protein 4 is involved in the urinary excretion of hydrochlorothiazide and furosemide. J. Am. Soc. Nephrol..

[38] Ci L., Kusuhara H., Adachi M., Schuetz J.D., Takeuchi K., Sugiyama Y. (2007). Involvement of MRP4 (ABCC4) in the luminal efflux of ceftizoxime and cefazolin in the kidney. Mol. Pharmacol..

[39] Smeets P.H., van Aubel R.A., Wouterse A.C., van den Heuvel J.J., Russel F.G. (2004). Contribution of multidrug resistance protein 2 (MRP2/ABCC2) to the renal excretion of *p*-aminohippurate (PAH) and identification of MRP4 (ABCC4) as a novel PAH transporter. J. Am. Soc. Nephrol..

[40] Ekaratanawong S., Anzai N., Jutabha P., Miyazaki H., Noshiro R., Takeda M., Kanai Y., Sophasan S., Endou H. (2004). Human organic anion transporter 4 is a renal apical organic anion/dicarboxylate exchanger in the proximal tubules. J. Pharmacol. Sci..

[41] Motohashi H., Inui K. (2013). Organic cation transporter OCTs (SLC22) and MATEs (SLC47) in the human kidney. AAPS J..

[42] Hori R., Okamura N., Aiba T., Tanigawara Y. (1993). Role of P-glycoprotein in renal tubular secretion of digoxin in the isolated perfused rat kidney. J. Pharmacol. Exp. Ther..

[43] Narawa T., Itoh T. (2010). Stereoselective transport of amethopterin enantiomers by the proton-coupled folate transporter. Drug Metab. Pharmacokinet..

[44] Tahara H., Kusuhara H., Fuse E., Sugiyama Y. (2005). P-glycoprotein plays a major role in the efflux of fexofenadine in the small intestine and blood-brain barrier, but only a limited role in its biliary excretion. Drug Metab. Dispos..

[45] Miura M., Uno T., Tateishi T., Suzuki T. (2007). Pharmacokinetics of fexofenadine enantiomers in healthy subjects. Chirality.

[46] Sakugawa T., Miura M., Hokama N., Suzuki T., Tateishi T., Uno T. (2009). Enantioselective disposition of fexofenadine with the P-glycoprotein inhibitor verapamil. Br. J. Clin. Pharmacol..

[47] Cvetkovic M., Leake B., Fromm M.F., Wilkinson G.R., Kim R.B. (1999). OATP and P-glycoprotein transporters mediate the cellular uptake and excretion of fexofenadine. Drug Metab. Dispos..

[48] Dresser G.K., Bailey D.G., Leake B.F., Schwarz U.I., Dawson P.A., Freeman D.J., Kim R.B. (2002). Fruit juices inhibit organic anion transporting polypeptide-mediated drug uptake to decrease the oral availability of fexofenadine. Clin. Pharmacol. Ther..

[49] Nozawa T., Imai K., Nezu J., Tsuji A., Tamai I. (2004). Functional characterization of pH-sensitive organic anion transporting polypeptide OATP-B in humans. J. Pharmacol. Exp. Ther..

[50] Shimizu M., Fuse K., Okudaira K., Nishigaki R., Maeda K., Kusuhara H., Sugiyama Y. (2005). Contribution of OATP (organic anion-transporting polypeptide) family transporters to the hepatic uptake of fexofenadine in humans. Drug Metab. Dispos..

[51] Bailey D.G., Dresser G.K., Leake B.F., Kim R.B. (2007). Naringin is a major and selective clinical inhibitor of organic anion-transporting polypeptide 1A2 (OATP1A2) in grapefruit juice. Clin. Pharmacol. Ther..

[52] Matsushima S., Maeda K., Ishiguro N., Igarashi T., Sugiyama Y. (2008). Investigation of the inhibitory effects of various drugs on the hepatic uptake of fexofenadine in humans. Drug Metab. Dispos..

[53] Matsushima S., Maeda K., Hayashi H., Debori Y., Schinkel A.H., Schuetz J.D., Kusuhara H., Sugiyama Y. (2008). Involvement of multiple efflux transporters in hepatic disposition of fexofenadine. Mol. Pharmacol..

[54] Tahara H., Kusuhara H., Maeda K., Koepsell H., Fuse E., Sugiyama Y. (2006). Inhibition of oat3-mediated renal uptake as a mechanism for drug-drug interaction between fexofenadine and probenecid. Drug Metab. Dispos..

[55] Matsushima S., Maeda K., Inoue K., Ohta K.Y., Yuasa H., Kondo T., Nakayama H., Horita S., Kusuhara H., Sugiyama Y. (2009). The inhibition of human multidrug and toxin extrusion 1 is involved in the drug-drug interaction caused by cimetidine. Drug Metab. Dispos..

[56] Akamine Y., Miura M., Komori H., Saito S., Kusuhara H., Tamai I., Ieiri I., Uno T., Yasui-Furukori N. (2014). Effects of one-time apple juice ingestion on the pharmacokinetics of fexofenadine enantiomers. Eur. J. Clin. Pharmacol..

[57] Kusuhara H., Miura M., Yasui-Furukori N., Yoshida K., Akamine Y., Yokochi M., Fukizawa S., Ikejiri K., Kanamitsu K., Uno T. (2013). Effect of coadministration of single and multiple doses of rifampicin on the pharmacokinetics of fexofenadine enantiomers in healthy subjects. Drug Metab. Dispos..

[58] Jonker J.W., Merino G., Musters S., van Herwaarden A.E., Bolscher E., Wagenaar E., Mesman E., Dale T.C., Schinkel A.H. (2005). The breast cancer resistance protein BCRP (ABCG2) concentrates drugs and carcinogenic xenotoxins into milk. Nat. Med..

[59] Van Herwaarden A.E., Wagenaar E., Karnekamp B., Merino G., Jonker J.W., Schinkel A.H. (2006). Breast cancer resistance protein (Bcrp1/Abcg2) reduces systemic exposure of the dietary carcinogens aflatoxin B1, IQ and Trp-P-1 but also mediates their secretion into breast milk. Carcinogenesis.

[60] van Herwaarden A.E., Wagenaar E., Merino G., Jonker J.W., Rosing H., Beijnen J.H., Schinkel A.H. (2007). Multidrug transporter ABCG2/breast cancer resistance protein secretes riboflavin (vitamin B_2_) into milk. Mol. Cell. Biol..

[61] Wang L., McNamara P.J. (2012). Stereoselective interaction of pantoprazole with ABCG2. I. Drug accumulation in rat milk. Drug Metab. Dispos..

[62] Wang L., Leggas M., Empey P.E., McNamara P.J. (2012). Stereoselective interaction of pantoprazole with ABCG2. II. In vitro flux analysis. Drug Metab. Dispos..

[63] Zhang L., Schaner M.E., Giacomini K.M. (1998). Functional characterization of an organic cation transporter (hOCT1) in a transiently transfected human cell line (HeLa). J. Pharmacol. Exp. Ther..

[64] Moaddel R., Patel S., Jozwiak K., Yamaguchi R., Ho P.C., Wainer I.W. (2005). Enantioselective binding to the human organic cation transporter-1 (hOCT1) determined using an immobilized hOCT1 liquid chromatographic stationary phase. Chirality.

[65] Moaddel R., Yamaguchi R., Ho P.C., Patel S., Hsu C.P., Subrahmanyam V., Wainer I.W. (2005). Development and characterization of an immobilized human organic cation transporter based liquid chromatographic stationary phase. J. Chromatogr. B Analyt. Technol. Biomed. Life Sci..

[66] Thyss A., Milano G., Kubar J., Namer M., Schneider M. (1986). Clinical and pharmacokinetic evidence of a life-threatening interaction between methotrexate and ketoprofen. Lancet.

[67] Maiche A.G. (1986). Acute renal failure due to concomitant action of methotrexate and indomethacin. Lancet.

[68] Uwai Y., Saito H., Inui K. (2000). Interaction between methotrexate and nonsteroidal anti-inflammatory drugs in organic anion transporter. Eur. J. Pharmacol..

[69] Takeda M., Khamdang S., Narikawa S., Kimura H., Hosoyamada M., Cha S.H., Sekine T., Endou H. (2002). Characterization of methotrexate transport and its drug interactions with human organic anion transporters. J. Pharmacol. Exp. Ther..

[70] Uwai Y., Taniguchi R., Motohashi H., Saito H., Okuda M., Inui K. (2004). Methotrexate-loxoprofen interaction: Involvement of human organic anion transporters hOAT1 and hOAT3. Drug Metab. Pharmacokinet..

[71] Nozaki Y., Kusuhara H., Kondo T., Iwaki M., Shiroyanagi Y., Nakayama H., Horita S., Nakazawa H., Okano T., Sugiyama Y. (2007). Species difference in the inhibitory effect of nonsteroidal anti-inflammatory drugs on the uptake of methotrexate by human kidney slices. J. Pharmacol. Exp. Ther..

[72] Uwai Y., Honjo H., Iwamoto K. (2010). Inhibitory effect of selective cyclooxygenase-2 inhibitor lumiracoxib on human organic anion transporters hOAT1 and hOAT3. Drug Metab. Pharmacokinet..

[73] Honjo H., Uwai Y., Aoki Y., Iwamoto K. (2011). Stereoselective inhibitory effect of flurbiprofen, ibuprofen and naproxen on human organic anion transporters hOAT1 and hOAT3. Biopharm. Drug Dispos..

[74] Beorlegui B., Aldaz A., Ortega A., Aquerreta I., Sierrasesúmega L., Giráldez J. (2000). Potential interaction between methotrexate and omeprazole. Ann. Pharmacother..

[75] Suzuki K., Doki K., Homma M., Tamaki H., Hori S., Ohtani H., Sawada Y., Kohda Y. (2009). Co-administration of proton pump inhibitors delays elimination of plasma methotrexate in high-dose methotrexate therapy. Br. J. Clin. Pharmacol..

[76] Chioukh R., Noel-Hudson M.S., Ribes S., Fournier N., Becquemont L., Verstuyft C. (2014). Proton pump inhibitors inhibit methotrexate transport by renal basolateral organic anion transporter hOAT3. Drug Metab. Dispos..

[77] Ikemura K., Hamada Y., Kaya C., Enokiya T., Muraki Y., Nakahara H., Fujimoto H., Kobayashi T., Iwamoto T., Okuda M. (2016). Lansoprazole exacerbates pemetrexed-mediated hematologic toxicity by competitive inhibition of renal basolateral human organic anion transporter 3. Drug Metab. Dispos..

[78] Hamada Y., Ikemura K., Iwamoto T., Okuda M. (2018). Stereoselective inhibition of renal basolateral human organic anion transporter 3 by lansoprazole enantiomers. Pharmacology.

[79] Miura M., Tada H., Yasui-Furukori N., Uno T., Sugawara K., Tateishi T., Suzuki T. (2004). Pharmacokinetic differences between the enantiomers of lansoprazole and its metabolite, 5-hydroxylansoprazole, in relation to CYP2C19 genotypes. Eur. J. Clin. Pharmacol..

[80] Kim K.A., Kim M.J., Park J.Y., Shon J.H., Yoon Y.R., Lee S.S., Liu K.H., Chun J.H., Hyun M.H., Shin J.G. (2003). Stereoselective metabolism of lansoprazole by human liver cytochrome P450 enzymes. Drug Metab. Dispos..

[81] Kawase A., Yamamoto T., Egashira S., Iwaki M. (2016). Stereoselective inhibition of methotrexate excretion by glucuronides of nonsteroidal anti-inflammatory drugs via multidrug resistance proteins 2 and 4. J. Pharmacol. Exp. Ther..

[82] Niwa T., Murayama N., Yamazaki H. (2011). Stereoselectivity of human cytochrome p450 in metabolic and inhibitory activities. Curr. Drug Metab..

[83] Ekins S., Ecker G.F., Chiba P., Swaan P.W. (2007). Future directions for drug transporter modelling. Xenobiotica.

[84] Bhatia P., Kolinski M., Moaddel R., Jozwiak K., Wainer I.W. (2008). Determination and modelling of stereoselective interactions of ligands with drug transporters: A key dimension in the understanding of drug disposition. Xenobiotica.

